# Interplay between Affect and Arousal in Recognition Memory

**DOI:** 10.1371/journal.pone.0011739

**Published:** 2010-07-23

**Authors:** Ciara M. Greene, Pooja Bahri, David Soto

**Affiliations:** Centre for Neuroscience, Imperial College London, London, United Kingdom; L'université Pierre et Marie Curie, France

## Abstract

**Background:**

Emotional states linked to arousal and mood are known to affect the efficiency of cognitive performance. However, the extent to which memory processes may be affected by arousal, mood or their interaction is poorly understood.

**Methodology/Principal Findings:**

Following a study phase of abstract shapes, we altered the emotional state of participants by means of exposure to music that varied in both mood and arousal dimensions, leading to four different emotional states: (i) positive mood-high arousal; (ii) positive mood-low arousal; (iii) negative mood-high arousal; (iv) negative mood-low arousal. Following the emotional induction, participants performed a memory recognition test. Critically, there was an interaction between mood and arousal on recognition performance. Memory was enhanced in the positive mood-high arousal and in the negative mood-low arousal states, relative to the other emotional conditions.

**Conclusions/Significance:**

Neither mood nor arousal alone but their interaction appears most critical to understanding the emotional enhancement of memory.

## Introduction

Studies of emotional influences on memory have revolved around Tulving and Thompson's [1] influential encoding specificity hypothesis that memory is facilitated by the congruency of emotional states during encoding and retrieval. In addition, close links have been described between emotion and memory processes other than encoding. Current understanding indicates that variations in emotional arousal after an initial study phase may influence subsequent memory for previously studied information. Liu, Graham and Zorawski [2] reported that arousal enhanced memory for preceding emotionally-targeted stimuli, while Anderson, Wais and Gabrieli [3] found that arousal manipulation following memory encoding improved subsequent consolidation and recollection of past neutral items. Further, it has been proposed that arousal levels, irrespective of emotional mood valence, may be critical for feature binding in working memory [4] and long-term memory for word lists [5]. However, other evidence [6] has suggested that mood valence, independently of arousal, may account for the emotional modulation of memory performance and that independent neural areas may support the influence of arousal and emotional valence on memory [7]. Memory for arousing items may rely on a neural network involving the amygdala and the hippocampus, whilst memory for valenced information may be supported by a prefrontal-hippocampal network [7].

A fundamental limitation of prior studies on the emotional modulation of memory was the absence of independent manipulations of mood and arousal states within the same experimental protocol. It therefore remains unclear whether mood, arousal or their interaction is most significant in the modulation of memory. To elucidate this issue, we used music to induce specific emotional states in the participants following encoding of a series of neutral stimuli and assessed subsequent effects on recognition memory. Critically, musical induction of emotion was manipulated independently in the mood and arousal dimensions. Music has been shown to be one of the best means of inducing emotion in an experimental setting [8], [9] and is therefore ideal for studying the influence of emotional state on subsequent memory.

If either arousal or mood states after memory encoding are key in determining subsequent recognition memory, we would expect to see a significant main effect of either mood or arousal on memory performance. However, if the interplay between the levels of arousal and the quality of the affective state is most critical then we hypothesise that a significant interaction effect between mood and arousal on recognition memory ought to be observed.

## Methods

### Participants

24 healthy participants (11 female) aged between 18 and 30 (mean = 22.63, SD = 2.55), with normal or corrected-to-normal vision, were recruited by means of an advertising campaign and were paid £10 for their participation. This research was approved by the Hammersmith and Queen Charlotte's & Chelsea Research Ethics Committee, and all participants provided informed written consent.

### Apparatus

Stimuli were presented using E-Prime 2.0 software (Psychology Software Tools, 2002) on a DELL laptop with 1280×800 screen resolution.

### Task and Procedure

#### Music selection

Participants listened to a series of short musical extracts and rated how each piece of music made them feel by placing a mark on a chart representing the dimensions of mood and arousal. The chart took the form of a large cross in which the vertical line denoted arousal, on a scale going from high (‘alert/energised’) to low (‘relaxed’) while the horizontal line represented mood on a continuum from ‘positive mood’ to ‘negative mood’. The pieces of music were drawn from a wide variety of genres (e.g. classical, jazz, blues, rock, heavy metal, electronica) with the sole restriction that they be instrumental pieces. The music selection process continued until participants had assigned at least one piece of music to each of the 4 quadrants, representing the 4 mood/arousal conditions.

#### Assessment of emotional states

Prior to the experimental task (see below), participants rated their mood (positive – negative) and arousal levels (high – low) on Visual Analogue Scales (VAS). The VAS consists of a 10cm horizontal line representing a continuum between the two extremes of each emotional variable, denoted by labels at either end of the line. Participants were asked to mark a point on the line to illustrate their position on the mood and arousal continua. Numerical scores of mood and arousal were calculated by measuring the distance between the participant's mark and the beginning of the line.

#### Study phase and recognition memory test

**Figure 1:**
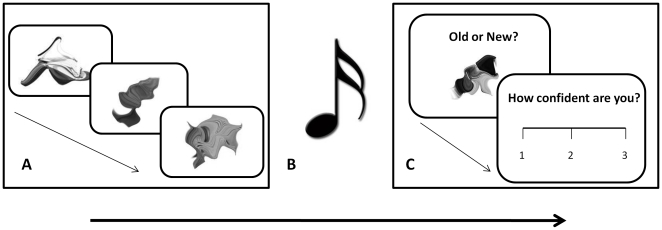
Experimental timeline. Four blocks of the experiment were conducted, each consisting of 3 phases. (A) Study phase; 20 abstract shapes were viewed for 5 seconds each. (B) Music exposure phase; music appropriate to the mood/arousal condition was played for 5 minutes. (C) Test phase; 20 old and 20 new shapes were presented. An old/new judgement was made, followed by confidence rating.

During the study phase of the task, participants were presented with a series of 20 randomly selected monochrome abstract shapes and were instructed to remember them in preparation for a subsequent recognition test. Music was not played during the study phase. The shapes were generated from bitmap images by custom software programmed in MATLAB (see Figure 1 for sample shapes and an experimental timeline). Each image had a resolution of 300×300 pixels and was presented centrally on a white screen at a viewing distance of approximately 50cm. Stimuli was presented for 5 seconds each with an inter-stimulus interval of 1 second. The study phase was followed by 5 minutes of music exposure. The participants were asked to pay attention to the music and, in order to maximise any emotional induction, were encouraged to derive thoughts and memories congruent with the emotional content of the music (see [9]). Following the music presentation, participants again rated their mood and arousal on VAS.

A recognition test was then performed. Each trial consisted of the presentation of an abstract shape followed by a 1 second inter-stimulus interval. 20 old (previously studied) shapes and 20 novel shapes were presented singly in random order. Participants were asked to indicate by a mouse click whether the shape was old (left click) or new (right click). Following the old/new response, participants were required to rate their confidence in the old/new judgement on a scale from 1 to 3, where 1 = not very confident, 2 = fairly confident and 3 = very confident. The confidence rating scale was displayed onscreen until a response was recorded. Participants were instructed to be as accurate as possible in their responses. Four blocks of the study and recognition tasks were performed, in which the music exposure following the study phase was varied to account for each of the 4 mood/arousal conditions. The order of the four conditions was counterbalanced across participants.

## Results

VAS ratings of mood and arousal prior to music exposure were compared across the 4 experimental blocks. One-way repeated measures ANOVAs indicated no significant differences between conditions in preliminary mood (F(3,69) = 1.609, p>.05) or arousal ratings (F(3,69) = 1.132, p>.05), suggesting that arousal and mood returned to a similar state at the beginning of each experimental block. The effect of music listening on mood and arousal ratings before and after music exposure was then assessed.

### Mood ratings

**Figure 2:**
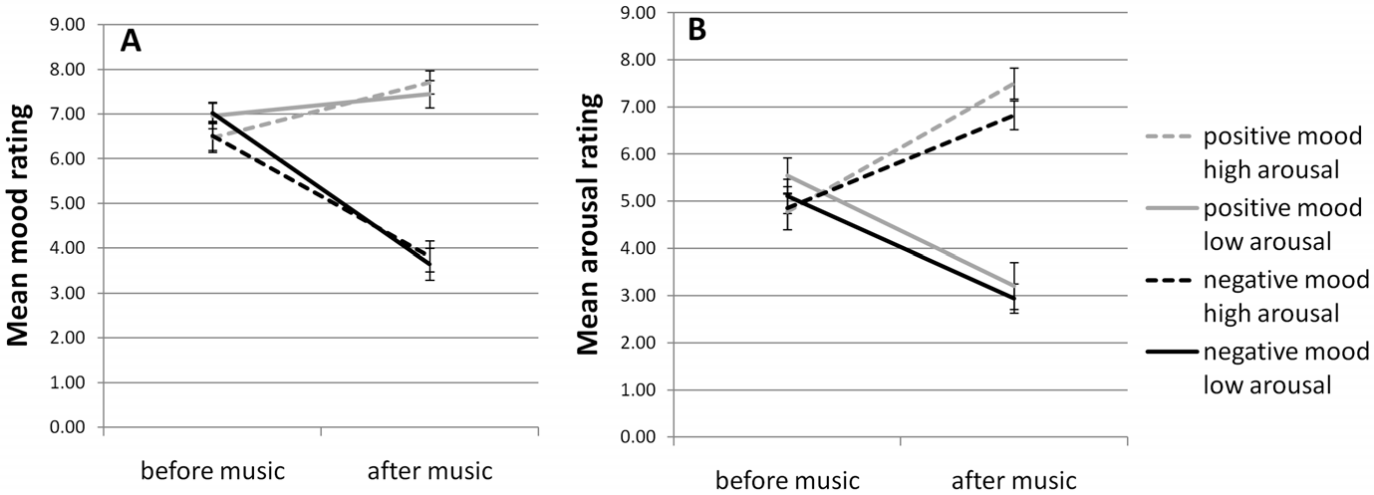
Subjective mood and arousal ratings before and after music presentation. (A) Mood ratings are seen to increase following presentation of music rated as more positive and decrease following music rated as negative. (B) Subjective arousal increased following music rated as highly arousing and decreased following music rated as less arousing. Error bars represent standard errors of the mean.

Mean mood ratings were entered into a 2×2×2 repeated measures ANOVA with mood dimension of the selected music (positive/negative), arousal dimension (high/low) and time (before/after the music). Main effects of the mood dimension of the music (F(1,23) = 70.909, p<.001, η^2^ = .75) and time (F(1,23) = 40.422, p<.001, η^2^ = .64) were discarded in the presence of a significant interaction between the mood dimension of the music condition and time (F(1,23) = 75.364, p<.001, η^2^ = .77): mood ratings increased after exposure to music rated as positive and decreased after music presentation rated as more negatively valenced (see Figure 2A). There was no main effect of the arousal dimension of the music on mood ratings (F(1,23) = .637, p>.05).

### Arousal ratings

Arousal ratings were highest following exposure to music rated as more arousing (F(1,23) = 55.197, p<.001, η^2^ = .71). There was no main effect on arousal ratings as a function of the mood elicited by the music (F(1,23) = 1.262, p>.05). There was no effect of time (F(1,23) = .048, p>.05). As expected, there was a significant interaction between the arousal dimension of the music and time (F(1,23) = 55.965, p<.001, η^2^ = .71) such that subjective arousal ratings increased following music rated as highly arousing and decreased with music rated as less arousing (see Figure 2B).

The above data confirm that music exposure was effective in inducing changes in mood and arousal states.

### Recognition memory performance

**Figure 3:**
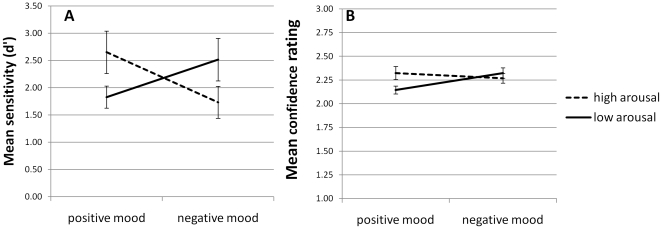
Sensitivity to old/new differences and confidence ratings in the four mood/arousal conditions. (A) The greatest sensitivity (d′) was observed during the positive mood/high arousal and negative mood/low arousal conditions. (B) A similar pattern was observed in ratings of confidence in old/new judgements.

Signal detection theory was used to calculate d′, a measure of sensitivity to old/new differences in all four mood/arousal conditions. The proportion of correct discriminations (hits) and proportion of false alarms (FA) were computed considering the presence of a new item as ‘signal present’ and an old item as ‘signal absent’. Thus, responding ‘new’ to a new item was labelled as a ‘hit’, whereas responding ‘new’ to an old item was labelled as a ‘false alarm’. In this way, we obtained the probability of hits – P(H) – and the P(FA) to calculate d′. Sensitivity (d′) scores for each participant were entered into a 2 (positive mood/negative mood)×2 (high arousal/low arousal) repeated measures ANOVA. No main effect of mood (F(1,23) = .315, p>.05) or arousal (F(1,23) = .008, p>.05) were observed, although there was a significant interaction effect (F(1,23) = 10.83, p<.001, η^2^ = .32). Paired t-tests confirmed that sensitivity was greater in the positive mood/high arousal case relative to the positive mood/low arousal condition (t(23) = 2.62, p<.05). The opposite pattern arose in the negative mood case where low arousal improved sensitivity relative to the high arousal condition (t(23) = 2.23, p<.05; see Figure 3A).

### Mean confidence ratings

**Figure 4:**
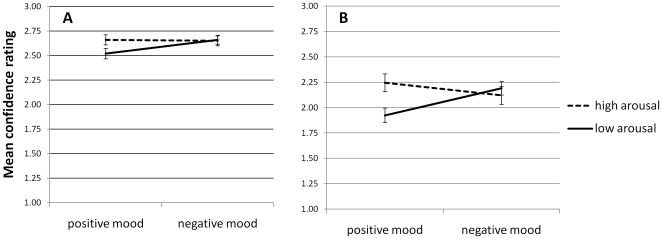
Confidence ratings during (A) ‘correct rejection’ and (B) ‘hit’ trials. The lowest confidence ratings were observed during the positive mood/low arousal conditions.

These were entered into a 2×2 ANOVA with mood and arousal as the independent variables. There was no main effect of arousal (F(1,23) = 3.832, p>.05). A main effect of mood was observed (F(1,23) = 5.137, p<.05, η^2^ = .18) which we discard in the presence of a significant interaction between mood and arousal (F(1,23) = 9.7, p<.05 η^2^ = .3; see Figure 3B), whereby the lowest confidence was reported during the positive mood/low arousal condition. A similar interaction was observed when the confidence ratings were analysed for correct rejections only (i.e. identifying an ‘old’ item as old; (F(1,23) = 12.21, p<.05, η^2^ = .35) and a trend in the same direction was apparent in the case of hit responses (F(1,23) = 3.65, p = .069). These data are illustrated in Figure 4.

## Discussion

Prior work on the role of emotion in influencing memory has failed to adequately distinguish between the effects of arousal and mood, frequently conflating them into a single measure of ‘positive’ versus ‘negative’ arousal (e.g., [2], [3], [5]). In addition, the design of many studies addressing this issue has called for the inclusion of arbitrary ‘neutral’ condition to act as a benchmark against which the effects of emotional manipulation can be measured [2], [9]. The definition of a ‘neutral’ emotional state is inherently ambiguous since a neutral emotional quality is only relative within the continuum of emotion state.

Here we induced affective states that varied independently in self-reported mood and arousal. We found enhanced memory sensitivity during positive mood/high arousal and negative mood/low arousal conditions. These data demonstrate for the first time that the interaction between mood and arousal states is critical in the modulation of memory. The fact that confidence ratings varied accordingly with mood valence and arousal suggests that emotion modulates recollection rather than simply enhancing a sense of familiarity.

The finding of recognition memory enhancement in the positive mood/high arousal condition might be predicted from studies showing that positive affect is associated with improved cognitive abilities including creative problem solving [10], spatial processing [11], [12] associative memory [9] and broadening of attention in health and disease [9], [13]. However, a positive affective state by itself was not sufficient to improve recognition memory since the positive affect/low arousal state was not associated with improved performance. The memory enhancement in the negative mood/low arousal state may seem counterintuitive, but this is not the first study to describe a performance benefit of this ‘melancholic’ state; Jefferies *et al.* [14] found that this combination of low arousal and negative mood improved selective attention to a rapid serial visual presentation. Other studies have reported that negative mood states may lead to a more detailed, local form of processing being adopted [15], [16]. We speculate that ‘melancholic’ states may be associated with the formation of more finely detailed memory representations.

Evidence suggests that there may be an optimal level of arousal for cognitive performance [17], [18]. Human and animal models indicate that the relationship between stress hormones (i.e. adrenaline, noradrenaline and cortisol) and cognition may follow an inverted U-shaped curve [19], [20], [21], and this relationship is mirrored in the neurocognitive response to catecholamine availability. There appears to be a critical level of dopamine and noradrenaline transmission for cognition, including memory, whereby an insufficiency or excess of neurotransmission can hamper performance [22], [23], [24]. Superior recognition performance was not observed here during the high arousal/negative mood condition, indicating that arousal alone does not provide a sufficient explanation of the results. We argue that an excess of noradrenergic activity in highly arousing states [25], [26] may be detrimental to recognition memory in the absence of a beneficial positive mood state. The current data demonstrate that the interplay between emotional factors is critical for cognitive processing, with a particular combination of mood and arousal being most important in the modulation of memory.
